# Mirabegron in Overactive Bladder and Its Role in Exit Strategy After Botulinum Toxin Treatment in Children

**DOI:** 10.3389/fped.2021.801517

**Published:** 2022-02-18

**Authors:** Denise Jia Yun Tan, Julia Weninger, Anju Goyal

**Affiliations:** Department of Paediatric Urology, Royal Manchester Children's Hospital, Manchester, United Kingdom

**Keywords:** Mirabegron therapy, overactive bladder, pediatrics, botox treatment, urinary incontinence, anticholinergics

## Abstract

**Purpose:**

Mirabegron is a recent addition to the management options of overactive bladder (OAB) in children. The purpose of this study was to ascertain the role of Mirabegron in the treatment algorithm of therapy-resistant OAB especially after botulinum toxin.

**Methods:**

Case notes of all children receiving Mirabegron between July 2017 and February 2020 were reviewed.

**Results:**

Forty one children (21 females, 20 males), mean age 12.6 [8–17] years old, commenced Mirabegron: 35 idiopathic OAB, 6 neuropathic OAB. The mean duration of treatment was 20.7 [3–45] months. In total 24 (59%) had Mirabegron after partial/no response to anticholinergics, and 17 (41%) patients had Mirabegron subsequent to botulinum toxin A (BtA) as an exit strategy. In total 35 (85%) patients had combination therapy (Mirabegron and anticholinergics), and 6 (15%) patients had Mirabegron only. Fourteen (34%) had complete response, 17 (41%) had partial response, and 10 (24%) had no response. Side effects were reported in 7 (17%) patients with discontinuation necessitated in 3.

**Conclusion:**

Mirabegron when used alone or in combination with anticholinergics resulted in complete/partial response in 76% of anticholinergic therapy-resistant OAB. In addition to being an important step in treatment escalation after no/partial response to anticholinergics, it has a crucial role in the exit strategy for recurring symptoms after BtA wears off.

## Introduction

Idiopathic overactive bladder (OAB) is a common lower urinary tract dysfunction (LUTD) in children and is characterized by the presence of urinary urgency accompanied with daytime urinary wetting and frequency (1). It may be associated with nocturia and/or nocturnal enuresis. OAB symptoms may also be associated with neuropathic bladder.

The overall prevalence of idiopathic OAB (IOAB) reported in children is between 5–17 % (2–4). Avoidance, lack of awareness and embarrassment of the symptoms presented could lead to the under-reporting of symptoms in children (5). The sequelae of uncontrolled symptoms of IOAB can have a negative impact on the children's emotional and behavioral well-being leading to social isolation, depression, and anxiety (6).

Anticholinergic medications such as oxybutynin, tolterodine, and solifenacin are the first line of medical treatment for children with OAB. These drugs act on M2 and M3 receptors in the bladder and antagonize the parasympathetic response to acetylcholine (7). The action of the drug on the receptors leads to the relaxation of bladder smooth muscle and helps toward reducing urine leakage. In children with OAB who have failed initial urotherapy and anticholinergic treatment, Mirabegron is an option. Mirabegron, a selective β3-adrenoreceptor agonist, is well-established in adult practice. The drug is not currently licensed for use in children (7). More recently, in 2021, Mirabegron is approved by the FDA for use in neurogenic detrusor overactivity (NDO) treatment in children (8). Mirabegron works by acting on the smooth muscles of the bladder, causing relaxation which results in the increase in bladder capacity without changing micturition pressure, voiding contraction, and post-void residual (7, 9). Mirabegron can be used as monotherapy or in combination with anticholinergics (7, 10, 11).

Botulinum toxin A (BtA) is an established treatment option in patients with refractory OAB. The drug prevents release of acetylcholine and adenosine 5-triphosphate from parasympathetic presynaptic terminations (2). This promotes a reversible desensitization and flaccid paralysis of the bladder muscle wall (2). If BtA is used in OAB, it is usually effective for 6–12 months (2). Many require repeated BtA injections 6–9-monthly for sustained relief of symptoms, and this requires general anesthesia in children. The exit strategy following BtA means that repeated BtA can possibly be avoided by commencing Mirabegron/anticholinergics when BtA wears off. We hypothesized that Mirabegron and/or anticholinergic medication has a good chance of being effective after BtA even if they were not helpful pre-BtA. Therefore, we investigated the role of Mirabegron as an option in the exit strategy after BtA.

The aim of our study is to:

1) To evaluate the effectiveness of Mirabegron in pediatric patients with refractory OAB.
2) To understand the role of Mirabegron in the medical treatment algorithm for children with OAB.
3) To review the adverse effects of Mirabegron in children.

## Methods

In our center, all children with OAB symptoms get a standard assessment with ultrasound urinary tract, uroflow studies, and bladder diary. The treatment algorithm starts with urotherapy and treatment of constipation if present, followed by introduction of anticholinergics as required. Oxybutynin is the first choice, and the dose is escalated to maximum tolerated. If there is partial/no response/adverse effects with oxybutynin, solifenacin is started at 5 mg and quickly escalated to 10 mg as required. If maximal anticholinergic therapy failed to produce an optimal response, we would escalate to the next step which is the addition of Mirabegron to anticholinergics. We prefer to commence Mirabegron, as combination therapy with anticholinergics and dose of anticholinergics was not reduced unless there were adverse effects on a higher dose.

However, prior to introduction of Mirabegron in 2017, intra-vesical botulinum toxin injection would be the next step after failure of anticholinergics.

We have 2 cohorts of patients:

Group 1 (treated with BtA before 2017) who had Mirabegron after BtA.
Group 2 who had Mirabegron after failure of anticholinergic therapy and did not have BtA before Mirabegron therapy.

Children who had BtA for OAB were advised to watch out for recurrence of symptoms, and at the first sign of onset of symptoms, they were started on anticholinergics and Mirabegron was added if required.

Case notes of all children receiving Mirabegron between July 2017 and February 2020 were reviewed retrospectively. Data on demographics, treatment details, dose, efficacy, and side effects were collected. Efficacy was assessed based on clinical response detailed in consultant-led clinic letters. Derived from the ICCS criteria with some modification, the response was documented as complete (>90% reduction of LUTD symptoms), partial (50%-90% reduction), and no response (<50% reduction) (1). The treatment efficacy was assessed on the reported patient or/and carers' description of response to treatment, including urinary frequency, urgency, day and/or night time wetting episodes, and impact on daily activities. The treatment efficacy response data were collected and interpreted by the clinician seeing the patient in clinic.

All children who were given Mirabegron had baseline tests including full blood count, renal and liver function test, blood pressure check, and 12-lead electrocardiogram (ECG). Routine monitoring of blood pressure was done 1 and 4 weeks after commencing Mirabegron treatment (7).

## Results

Between July 1, 2017, to February 29, 2020, 41 children (21 females), ranging from 8 to 17 years (mean 12.6 years), received Mirabegron treatment. Mirabegron was commenced at a dose of 25 mg and later increased to 50 mg as required. At the time of assessment, 27 children were on a 50-mg dose. Fourteen children were on 25 mg, and of these 7 had CR, 4 had PR, and 3 had NR. Of 7 children with NR/PR, the dose was not increased to 50 mg as there were side effects in 2, 1 wanted BtA, and others were still in the process of treatment escalation. Patient data were updated in April 2021. The mean treatment duration was 20.7 [range 3–45] months at the time of evaluation. The mean follow-up was 23.5 months. The primary diagnosis was IOAB in 35 (85%), whereas 6 (15%) had neuropathic etiology. Mirabegron was used in combination with anticholinergics in 35 (85%), 33 patients were on 10 mg solifenacin, and 2 were on 15 mg oxybutynin. The remaining 6 (15%) patients were on Mirabegron monotherapy, usually due to intolerance to anticholinergics.

### Response to Mirabegron Treatment

On combination therapy, 12 (34%) patients had complete response (CR) and 13 (37%) patients had partial response (PR). Ten (29%) patients had no response (NR) (Table 1). There were 6 children on monotherapy, and of these 2 (33%) had complete response and 4 (67%) had partial response. Overall, 31 (76%) children had partial/complete response to Mirabegron treatment (Table 1).

**Table 1:** The response of Mirabegron treatment in children.

	**Combination therapy**	**Monotherapy**	**Overall**
	**number (%)**	**number (%)**	**number (%)**
Total numbers	35 (85%)	6 (15%)	41 (100%)
Complete response (CR)	12 (34%)	2 (33%)	14 (34%)
Partial response (PR)	13 (37%)	4 (67%)	17 (41%)
Overall positive	25 (71%)	6 (100%)	31 (76%)
response			
No response (NR)	10 (29%)	0 (0%)	10 (24%)

### Mirabegron After BtA

Seventeen (41%) children (Group 1) had Mirabegron for return of symptoms after BtA. Fifteen received a combination therapy of Mirabegron and an anticholinergic, and 2 had Mirabegron alone. The Group 1 cohort had an average of 1.9 BtA sessions before Mirabegron. Seven had CR, 8 had PR, and 2 had NR. Only 4 needed further BtA after Mirabegron therapy. The mean duration between a patient receiving their last BtA and assessment of Mirabegron success was 44.5 months, thus excluding any residual effect from BtA. Our algorithm is to start back on anticholinergic as soon as there are first signs of symptoms of OAB after BtA and add Mirabegron if symptoms are only partially responding to anticholinergic.

### Reported Side Effects of Mirabegron

The adverse effects reported in 7 patients included headaches (*n* = 2), nausea (*n* = 1), dizziness (*n* = 1), disturbed vision (*n* = 1), chest pain (*n* = 1), abdominal pain (*n* = 1), high blood pressure (*n* = 1), and being generally unwell (*n* = 1). Discontinuation of Mirabegron treatment was necessitated in 3 (7%) patients: one patient experienced nausea and vomiting, another patient was feeling generally unwell, and one suffered from high blood pressure (136/90).

## Discussion

Mirabegron, a β3-adrenoceptor-selective agonist, is not currently licensed for use in children, although few available studies in children have demonstrated promising results (7, 11, 12). However, Mirabegron is recently approved by the FDA for use in neurogenic bladder treatment in children (8). In adult patients, NICE guidelines recommend the use of Mirabegron after first-line treatments such as anticholinergics have failed or there are adverse effects of drug treatment (13). A systematic review and meta-analysis conducted by Kelleher et al. (10) demonstrated that the outcome of Mirabegron use in adults with OAB was better than that of placebo at reducing LUTD symptoms and as effective as most antimuscarinic therapy with fewer anticholinergic side effects as dry mouth, constipation, and urinary retention. Blais et al. (7) conducted a prospective pilot study on 58 pediatric patients who had not responded to medical therapy with at least two different anticholinergics. The study concluded that 58 refractory OAB patients who underwent 11.5 months of Mirabegron monotherapy had significant improvement in bladder capacity and up to 90% of patients showed improvement in LUTD symptoms (7). In our cohort of refractory OAB, 31 (76%) children reported improvement in their symptoms after taking Mirabegron, either alone or in combination with anticholinergics. While our success rates are not as high as the study by Blais et al. (7), it is clear that Mirabegron has a role in the management of children with refractory OAB.

### Mono- and Combination Therapy

Mirabegron acts on β3 receptors and anticholinergics on Ach receptors; therefore, it would be logical to use them in combination to achieve maximal bladder relaxation. The meta-analysis by Kelleher et al. (10) demonstrated that combination therapy of Mirabegron 25 mg (or 50 mg) with solifenacin 5 mg was more effective than Mirabegron alone. According to them, solifenacin plus Mirabegron in combination can be an effective treatment in patients who are not responding adequately to anticholinergics alone and potentially reduces the number of patients who need invasive treatment options like intravesical BtA (10).

Another prospective off-label study was conducted by Morin et al. (12) on 35 refractory OAB pediatric patients, and Mirabegron was given as dual therapy along with anticholinergics for 16.4 months. The study demonstrated improvement in all patients with a median increase bladder capacity of 24%, 66% had partial response, and 34% patients were completely dry after combination therapy (12). It is our practice to prefer combination therapy to Mirabegron monotherapy in refractory OAB children, and 85% of our patients were started on combination therapy. The reason for monotherapy was intolerance to anticholinergics.

Fryer et al. (11) reported their experience of Mirabegron in 68 refractory OAB children, mostly used as monotherapy. They reported that in their cohort 31 (46%) discontinued Mirabegron due to either no improvement or side effects. Of the 37 children who continued Mirabegron for more than 6 months, 70% showed improvement in symptoms. Monotherapy was taken by 32 patients and combination therapy by 5 (Solifenacin) (11). Fryer et al. (11) suggested to consider combination therapy as soon as monotherapy does not improve symptoms.

### Exit Strategy After BtA Treatment

When OAB symptoms are refractory to anticholinergics/Mirabegron, BtA is the next option. While BtA is a well-recognized therapeutic option, there is not a clear pathway as to what happens after it wears off. For recurrence of symptoms after BtA, the options are to consider either repeat BtA or anticholinergics. Ingham et al. (14) reported that 92% of children have recurrence of OAB symptoms after initial BtA and 44% require repeat BtA. In our experience, complete long-term resolution after single BtA is rare.

While there is recurrence of detrusor overactivity 6–9 months after BtA, there is a long-term improvement in baseline detrusor function which makes oral anticholinergics and/or Mirabegron effective even though it has not been so before BtA. In our practice, anticholinergic/Mirabegron combination therapy is an important rescue therapy after BtA.

In our cohort (group 1), 17 children were given Mirabegron for recurrence of symptoms after BtA. Of these, 7 had complete response, 8 had PR, and 2 had no response (1 discontinued due to side effects). Only 4 (24%) of the 17 children required repeat BtA as opposed to 44% reported in other studies (14). The BtA treatment dose for children 5 units per kg, maximum of 150 units first time. If the patient continues to demonstrate a suboptimal response after the initial treatment dose, a higher dose may be required at 10 units per kg, maximum 300 units.

### Adverse Effects

Mirabegron is a well-tolerated drug. However, side effects have been reported. The commonly reported side effects of Mirabegron use in adults are dry mouth, headaches, and urinary retention (10, 13, 15). In children, reported side effects include xerostomia, headaches, dizziness, seizures, nausea, vomiting, rash, abdominal pain, chest pains, heart rate, and blood pressure changes (7, 11, 12). The side effects of Mirabegron may necessitate discontinuation of treatment. In our study, only 7% of the children discontinued treatment due to adverse effects. Fryer et al. (11) also reported that 10% of children discontinued Mirabegron due to adverse effects which include headaches, nausea, vomiting, dry mouth, seizures, and rash. Blais et al. (7) reported side effects in 9% of the children, and discontinuation of treatment was necessary in 5%.

In adults, cardiovascular side effects such as hypertension and prolonged QT interval on ECG are one of the main concerns under Mirabegron treatment (9). Extra caution should be taken in patients who have a history of hypertension and long QT syndrome (13). In our cohort, all children had ECG conducted before the commencement of Mirabegron treatment to check for QT prolongation and had blood pressure monitoring after commencement. All our patients had normal ECG/QTc intervals and normal blood pressure prior to treatment. After commencement, 1 patient had to stop therapy due to high blood pressure. Other side effects experienced by our patient cohort were headaches, nausea, dizziness, vision disturbances, chest pain, and abdominal pain.

### Stepwise Approach to OAB

The stepwise medical management of IOAB in children starts with anticholinergics as first line after urotherapy. Based on the limited literature available, Mirabegron represents a viable step in the treatment algorithm of children with refractory OAB. In children who have inadequate response to anticholinergics but are able to tolerate it, we usually add Mirabegron as combination therapy. If they have partial or no response to this, then BtA is the next escalation in treatment. When BtA effect wears off and symptoms recur, we again start medical treatment with anticholinergic and/or Mirabegron. In case of partial/no response, repeat BtA is considered.

Our study has several limitations. Patient data collected from medical records detailing description of symptom reduction can be challenging for subjective interpretation, specifically for partial and no response to Mirabegron treatment. Furthermore, our study is limited by a relatively small sample size. In our clinical experience, getting children to provide meaningful bladder diary data on a consistent basis for evaluation outside of research setting is very difficult, mainly because of poor patient compliance.

## Conclusion

Mirabegron alone or in combination improved LUTD symptoms in 31 (76%) of 41 patients with refractory OAB. It is effective and safe for children, mostly used in combination with anticholinergics as next step in treatment escalation after no/partial response to anticholinergics. Our study highlights the role of Mirabegron in the exit strategy after BtA. Mirabegron helps in reducing the need for repeated BtA injections. Further research into the objective measurement of effect of Mirabegron therapy with use of frequency–volume charts would be a way forward to substantiate our initial results.

## Data Availability Statement

The original contributions presented in the study are included in the article/Supplementary Material, further inquiries can be directed to the corresponding author/s.

## Author Contributions

DT and AG collected, analyzed results, and draft the manuscript. JW helped with the analysis, interpretation of results, and literature search. All authors read and approved the final manuscript.

## Conflict of Interest

The authors declare that the research was conducted in the absence of any commercial or financial relationships that could be construed as a potential conflict of interest.

## Publisher's Note

All claims expressed in this article are solely those of the authors and do not necessarily represent those of their affiliated organizations, or those of the publisher, the editors and the reviewers. Any product that may be evaluated in this article, or claim that may be made by its manufacturer, is not guaranteed or endorsed by the publisher.

## References

[1] Austin PF Bauer SB Bower W Chase J Franco I Hoebeke P. The standardization of terminology of lower urinary tract function in children and adolescents: Update report from the standardization committee of the International Children's Continence Society. Neurourol Urodynam. (2006) 35:471–81. 10.1002/nau.22751 25772695

[2] Oliveria RG Ubirajara BJ. Overactive bladder in children. Eur Med J. (2018) 3:70–7. Available online at: https://www.emjreviews.com/urology/article/overactive-bladder-in-children/

[3] Kajiwara M Inoue K Kato M Usui A Kurihara M Usui T. Nocturnal enuresis and overactive bladder in children: an epidemiological study. Int J Urol. (2006) 13:36–41. 10.1111/j.1442-2042.2006.01217.x 16448430

[4] Chung JM Lee SD Kang DI Kwon DD Kim KS Kim SY. Prevalence and associated factors of overactive bladder in Korean children 5–13 years old: a nationwide multicenter study. Urology. (2009) 73:63–7. 10.1016/j.urology.2008.06.063 18829077

[5] Chiozza ML Bernardinelli L Caione P Del Gado R Ferrara P Giorgi PL. An Italian epidemiological multicentre study of nocturnal enuresis. Br J Urol. (1998) 81:86–9. 10.1046/j.1464-410x.1998.00015.x 9634027

[6] Landgraf JM Abidari J Cilento BG Cooper CS Schulman SL Ortenberg J. Coping, commitment, and attitude: quantifying the everyday burden of enuresis on children and their families. Pediatrics. (2004) 113:334–44. 10.1542/peds.113.2.334 14754946

[7] Blais AS Nadeau G Moore K Genois L Bolduc S. Prospective pilot study of Mirabegron in pediatric patients with overactive bladder. Eur Urol. (2016) 70:9–13. 10.1016/j.eururo.2016.02.007 26876327

[8] US Food Drug Administration (2021). FDA Approves New indication for Drug to Treat Neurogenic Detrusor Overactivity in Pediatric Patients. Available online at: https://www.fda.gov/news-events/press-announcements/fda-approves-new-indication-drug-treat-neurogenic-detrusor-overactivity-pediatric-patients. (accessed December 4, 2021).

[9] White WB Chapple C Gratzke C Herschorn S Robinson D Frankel J. Cardiovascular safety of the β3-adrenoceptor agonist Mirabegron and the antimuscarinic agent solifenacin in the SYNERGY trial. J Clin Pharmacol. (2018) 58:1084–91. 10.1002/jcph.1107 29645285

[10] Kelleher C Hakimi Z Zur R. Efficacy and tolerability of Mirabegron compared with antimuscarinic monotherapy or combination therapies for overactive bladder: a systematic review and network meta-analysis. Eur Urol. (2018) 74:324–33. 10.1016/j.eururo.2018.03.020 29699858

[11] Fryer S Nicoara C Dobson E Griffiths M McAndrew HF Kenny SE. Effectiveness and tolerability of Mirabegron in Children with overactive bladder: a retrospective pilot study. J of Paed Surg. (2020) 55:316–8. 10.1016/j.jpedsurg.2019.10.044 31759655

[12] Morin F Blais AS Nadeau G Moore K Genois L Bolduc S. Dual therapy for refractory overactive bladder in children: a prospective open-label study. J Urol. (2016) 197:1158–63. 10.1016/j.juro.2016.11.101 27914999

[13] NICE UK (2013). Mirabegron for treating symptoms of overactive bladder. Available online at: https://www.nice.org.uk/guidance/TA290/chapter/1-Guidance. (accessed January 30, 2021)

[14] Ingham J Angotti R Lewis M Goyal A. Onabotulinum toxin A in children with refractory idiopathic overactive bladder: medium-term outcomes. J Pediatr Urol. (2019) 15:32.e1–32.e5. 10.1016/j.jpurol.2018.08.007 30224301

[15] Khullar V Amarenco G Angulo JC Cambronero J Høye K Milsom I. Efficacy and tolerability of mirabegron, a β^3^-adrenoreceptor agonist, in patients with overactive bladder: results from a randomised european-australian phase 3 trial. Eur Urol. (2013) 63:283–95. 10.1016/j.eururo.2012.10.016 23182126

